# Virgin Olive Oil Extracts Reduce Oxidative Stress and Modulate Cholesterol Metabolism: Comparison between Oils Obtained with Traditional and Innovative Processes

**DOI:** 10.3390/antiox9090798

**Published:** 2020-08-27

**Authors:** Carmen Lammi, Nadia Mulinacci, Lorenzo Cecchi, Maria Bellumori, Carlotta Bollati, Martina Bartolomei, Carlo Franchini, Maria Lisa Clodoveo, Filomena Corbo, Anna Arnoldi

**Affiliations:** 1 Department of Pharmaceutical Sciences, University of Milan, 20133 Milan, Italy; carlotta.bollati@unimi.it (C.B.); martina.bartolomei@unimi.it (M.B.); anna.arnoldi@unimi.it (A.A.); 2 Department of Neuroscience, Psychology, Drug and Child Health, Pharmaceutical and Nutraceutical Section, University of Florence, 50019 Florence, Italy; nadia.mulinacci@unifi.it (N.M.); lo.cecchi@unifi.it (L.C.); maria.bellumori@unifi.it (M.B.); 3 Department of Pharmacy-Pharmaceutical Sciences, University Aldo Moro Bari, 70125 Bari, Italy; carlo.franchini@uniba.it (C.F.); filomena.corbo@uniba.it (F.C.); 4 Interdisciplinary Department of Medicine, University Aldo Moro Bari, 70125 Bari, Italy; marialisa.clodoveo@uniba.it

**Keywords:** antioxidant, HepG2 cells, EVOO extract, IOC methods, LDLR, PCSK9

## Abstract

This study was aimed at demonstrating the substantial equivalence of two extra virgin olive oil samples extracted from the same batch of Coratina olives with (OMU) or without (OMN) using ultrasound technology, by performing chemical, biochemical, and cellular investigations. The volatile organic compounds compositions and phenolic profiles were very similar, showing that, while increasing the extraction yields, the innovative process does not change these features. The antioxidant and hypocholesterolemic activities of the extra virgin olive oil (EVOO) phenol extracts were also preserved, since OMU and OMN had equivalent abilities to scavenge the 1,1-diphenyl-2-picrylhydrazyl (DPPH) and 2,2′-azino-bis(3-ethylbenzothiazoline-6-sulfonic acid) diammonium salt (ABTS) radicals in vitro and to protect HepG2 cells from oxidative stress induced by H_2_O_2_, reducing intracellular reactive oxygen species (ROS) and lipid peroxidation levels. In addition, by inhibiting 3-hydroxy-3-methylglutarylcoenzyme a reductase, both samples modulated the low-density lipoprotein receptor (LDLR) pathway leading to increased LDLR protein levels and activity.

## 1. Introduction

New lifestyles, higher incomes, and consumer awareness are creating consumer demand for high-quality, diverse, and innovative food products. One of the goals of the European Union is to guarantee food safety and security in a changing world, under the effects of climate change, resource paucity, and population dynamics. The development of innovative technologies and sustainable business models for food systems is a crucial factor for boosting the competitiveness of the European industry [1]. European research programs supporting new technologies and innovative products are continuously introduced in the food market. However, in respect to most of the other industrial sectors, food consumers are not very favorable to modify their customs and to accept innovations, partly due to a phenomenon known as neophobia that is the rejection of new or unfamiliar foods [2].

Our interdisciplinary research team is applying an innovative and sustainable process method to produce high-quality, cost-effective, and resource-efficient extra virgin olive oil (EVOO), employing an emerging technology based on the simultaneous treatment of olive paste both with ultrasound and heat-exchange [3,4,5]. The application of ultrasound on olive oil extraction has been studied by many authors in different olive growing areas [3,4,5,6,7,8,9,10,11,12,13,14,15,16]. Ultrasound is sound waves with frequencies higher than the upper audible limit of human hearing (greater than 20 kHz) [17,18]. Due to the mechanical effects of the sound waves within the olive paste, it is possible to eliminate the malaxation, the only batch phase in the continuous extraction process. When the ultrasound wave is propagating in the crushed olive paste, it determines an alternation of positive and negative pressures inside it. When the negative pressure values are below the water vapor pressure in the olive paste, it undergoes a phase change from liquid to gas, forming cavities containing steam and giving rise to the phenomenon of cavitation. Cavitation is a physical phenomenon consisting of the formation of cold vapor bubbles inside a fluid that then implode, producing shock waves. If implosion occurs near the cell wall of the olive fruit, it generates a liquid microjet that breaks the wall, freeing oil and minor compounds [16].

This is a ready-to-market technological solution, combined with practices and management strategies to help olive oil producers to increase the production yields [13], while preserving the healthy properties of the oil, improving the process efficiency, and valorizing the wastes [14,15,16]. The application of this strategic innovation may occur on a large scale, although only if this type of EVOO is well accepted by the market and a premium price is recognized for its high quality, overcoming the natural neophobia that accompanies every innovation in a traditional food sector, such as EVOO [17,18]. In fact, the consumer may percept a food produced with an innovative process as less natural and less good than a traditional one. In order to improve the diffusion of this strategic innovation in the oil sector, it is thus mandatory to demonstrate the substantial equivalence of the innovative and traditional products.

This investigation was conducted on two EVOO samples extracted from the same batch of fruits of the cultivar Coratina with (OMU) or without (OMN) using the ultrasound technology. The first objective of the study was to show that the two samples had comparable volatile organic compounds (VOCs) profiles, because this profile is more and more used in chemical/statistical models for supporting the panel test in virgin olive oil classification [19]. The VOCs profile was determined using a validated solid-phase microextraction (HS-SPME) followed by gas chromatography (GC) coupled with mass spectrometry (MS) (HS-SPME-GC-MS) method for the reliable quantification of 73 VOCs based on the use of the Multiple Internal Standard Normalization (MISN) and 73 calibration lines built with authentic external standards [19,20].

The second objective was to verify the similarity of the phenolic profiles since phenols provide relevant health benefits. In particular, the European Union has recently approved the health claim that “olive oil polyphenols contribute to the protection of blood lipids from oxidative stress” and that “the claim may be used only for olive oil which contains at least 5 mg of hydroxytyrosol and its derivatives (e.g., oleuropein complex and tyrosol) per 20 g of olive oil” [21,22]. The detailed phenolic profiles were determined by using the International Olive Council (IOC) official method for total phenols as well as a recently validated hydrolytic procedure for total hydroxytyrosol (HT) and tyrosol (Tyr) [21].

Furthermore, another objective was to prove that the new process does not modify or impair the biological properties of the EVOO phenols in term of the antioxidant and hypocholesterolemic properties. The antioxidant activity was evaluated in vitro by employing the 1,1-diphenyl-2-picrylhydrazyl (DPPH) radical assay as well as the 2,2′-azino-bis(3-ethylbenzothiazoline-6-sulfonic acid) diammonium salt (ABTS) assay, whereas the capacity to reduce the level of intracellular radical oxygen species (ROS) and lipid peroxidation was measured in HepG2 where the oxidative stress was induced by H_2_O_2_. Finally, an in-depth investigation was performed on the capacity of both extracts to modulate cholesterol metabolism, by carrying out molecular and functional experiments. In fact, in a previous work [23], we had shown that OMN is able to modulate, in a favorable way, this metabolic pathway. For this reason, OMN was used as reference extract and the evaluation of the OMU ability to modulate the LDLR-pathway was carried out in order to assess how the ultrasound process affects the biological activity of the new extract.

## 2. Materials and Methods

### 2.1. Chemicals

All chemicals employed are from commercial sources. See Supplementary Materials for further details.

### 2.2. Sonicated Extra Virgin Olive Oil

The oils were produced from olives from the cultivar Coratina cultivated in Apulia in the period 2017–2018 in industrial plants producing olive oil (1500–3000 kg/h) located in Apulia Region (Frantoio MIMI). The extraction line was equipped with a two-phase centrifugal system for oil separation. The mill had a hammer crusher and the malaxers were hermetically closed. The homogeneous batches of olives were divided into samples of 800 kg each. After the selection and the washing, the olives were crushed. The crushed olive paste was then passed into the Sono Heat Exchanger (SHE) and then fell into the malaxer. The SHE was characterized by a work capacity equal to 1500 kg/h and was equipped with 56 transducers (100 watt and 31 kHz) able to transfer a specific energy equal 18,000 kJ/kg [5]. Moreover, the SHE was able to cool and heat the olive paste as a function of the environmental temperature in order to keep the temperature constant. Malaxers were used as buffers to continuously feed the decanter; the time of malaxation was set at 0 min for the sonicated samples and equal to 30 min for the traditional samples. The resulting EVOO was collected, filtered, and stored in dark bottles (at 15 °C) until chemical analysis.

The OMN and OMU samples’ production conditions are identical except for the sonication phase present before the crusher in the OMU sonicated samples and absent in the OMN unsonicated samples.

### 2.3. HS-SPME-GC-MS Analysis of Volatile Organic Compounds

The composition of the EVOO headspaces were characterized using a HS-SPME-GC-MS method, following the same conditions already described in detail in previous studies [19,20]. The peak identification was based on the comparison of the GC-MS parameters with those of authentic standards. The quantitation of each of the 73 identified VOCs was based on the use of 73 calibration lines built using authentic external standards, after normalization of peak areas using 9 suitable internal standards [19,20].

### 2.4. Analysis of Phenols in EVOO and Defatted Extracts

The extraction of phenols was carried out in triplicate with MeOH:H_2_O 80:20 *v*/*v* according to the IOC method [24] (IOC/T.20/Doc No. 29) and the analyses were carried out with a HP 1100 system (quaternary pump, Diode-Array Detection (DAD) detector, autosampler from Agilent Technologies, Santa Clara, CA, USA). The column was a SphereClone ODS (2), 5 μm, 250 × 4.6 mm; the elution solvents were acidified H_2_O by phosphoric acid (pH 2.0), CH_3_CN and MeOH and the applied gradient was in accordance with the IOC method [24].; for the quantitative analysis, the area values were collected at 280 nm and the results expressed as mg tyrosol/kg oil, using syringic acid as internal standard (ISTD).

The acid hydrolysis was applied to the extracts obtained as above (the hydrolyzed extracts were not used for the biological tests) and the sum of free and bound hydroxytyrosol and tyrosol was determined at 280 nm [21]. Briefly, the extract (300 μL) was added with 1.0 M H_2_SO_4_ (300 μL), then heated at 80 °C for 2 h and, after cooling, the solution was diluted with distilled water (400 μL). The analysis was carried out using a column, 150 × 3 mm (5 μm) RP18-Gemini (Phenomenex, CA, USA); the HPLC-DAD system was HP 1200 (Agilent Technologies, Santa Clara, CA, USA). The flow rate was 0.4 mL/min, the eluent A was H_2_O at pH 3.2 by HCOOH, and eluent B was acetonitrile. The linear applied gradient was from A 95% to 70% (5 min), then 5 min to A 50% and other 5 min to A 2% with a final plateau of 5 min. Total time of analysis and equilibration time were 22 min and 10 min respectively. Tyrosol was evaluated using the curve built with pure tyrosol (purity 98%); the hydroxytyrosol amount was evaluated using the same calibration line but applying a corrective factor for not overestimate the concentration: mg OH-tyrosol = mg tyrosol × 0.65 [25].

### 2.5. Phenolic Extracts for Testing on Cell Lines

For each oil, 50 g were exactly weighted and extracted by 150 mL of MeOH:H_2_O 80:20 *v*/*v* mixture and vigorously hand-shaken for some minutes, then the extraction was concluded with the aid of an ultrasound bath (10 min). The sample was centrifuged at 5000 rpm for 25 min and the solution recovered and filtered by polyvinylidene fluoride (PVDF) type 0.45 µm filter. The residual lipid was removed adding hexane (75 mL for twice), the defatted hydro-alcoholic solution was dried under vacuum at room temperature. The dry samples were dissolved in ethanol (in a flask of 10 mL) and the solution split in 10 vials in order to obtain 1 mL corresponding to 10 g of EVOO (these samples were analyzed as described in the previous paragraph). The solvent was removed from each vial by a flux of nitrogen to obtain 10 aliquots of the dried extract to be stored over time before the chemical and biological tests. By this way, each vial of OMN contained 5.6 ± 0.12 mg of dry weight and OMU 6.0 ± 0.09 mg of dry weight.

### 2.6. Proton Spectra of the Phenolic Extract

^1^H-NMR spectra of dry OMN and OMU were acquired as previously suggested [26] to investigate on the aldehydes derived by oleuropein and ligstroside. Briefly, one vial of each extract (obtained as above described from 10 g of EVOO) was dissolved adding 1 mL of CDCl_3_ and analyzed by 400 MHz instrument (Advance 400 from Bruker, Bremen, Germany).

### 2.7. DPPH Assay for Evaluating the In Vitro Radical Scavenging Activity

The DPPH assay to determine the antioxidant activity in vitro was performed by a standard method with slight modifications [27]. The DPPH solution (0.0125 mM in MeOH, 45 μL) was added to 15 μL of the OMN and OMU EVOO extracts at different concentrations (1.0 and 50.0 µg/mL). The reaction for scavenging the DPPH radicals was performed in the dark at room temperature (RT) and the absorbance was measured at 520 nm after 30 min incubation.

### 2.8. ABTS Assay for Evaluating the In Vitro Radical Scavenging Activity

Aliquots of 10 μL of OMN and OMU EVOO extracts (5.0, 10.0, 50.0 and 100.0 μL/mL) or trolox standards were added to individual wells of the assay plate provided from the kit (ABTS Antioxidant Assay Kit, Zen Bio, NC, USA), using the assay buffer as a negative control. Then, 20 μL of the myoglobin working solution was added to each well. To begin the assay, 100 μL of the ABTS solution per well was added and the absorbance at a wavelength of 405 nm was detected using the kinetic mode, for 30 min, through the Synergy H1 plate reader (Biotek, Bad Friedrichshall, Germany), later the reaction was stopped by adding 50 µL of Stop Solution and the absorbance at 405 nm was detected.

### 2.9. Cell Culture Conditions and Treatments

HepG2 cells, purchased from ATCC (HB-8065, ATCC from LGC Standards, Milan, Italy), were cultured following conditions already optimized [23]. More details are available on Supplementary Materials.

OMN and OMU extracts were tested separately. Briefly, each extract was dissolved in DMSO in order for preparing a stock solution of 50.0 mg/mL, which was diluted in order to reach the final concentration of 25.0 µg/mL in complete growth Doulbecco’s Modified Eagle Medium (DMEM). The final 0.05% concentration of DMSO was kept constant either in treated or control cells.

### 2.10. 3-(4,5-Dimethylthiazol-2-yl)-2,5-Diphenyltetrazolium Bromide (MTT) Assay

A total of 3 × 10^4^ HepG2 cells/well were seeded in 96-well plates and treated with 25.0, 50.0, 100.0 and 200.0 μg/mL of OMN and OMU samples, or vehicle (H_2_O) in complete growth media for 48 h at 37 °C under 5% CO_2_ atmosphere. MTT experiments have been performed following conditions previously optimized [23]. More details are available in Supplementary Materials.

### 2.11. Fluorometric Intracellular ROS Assay

For cells preparation, 3 × 10^4^ HepG2 cells/well were seeded on a 96-well plate overnight in growth medium. The day after, the medium was removed, 50 μL/well of Master Reaction Mix was added and the cells were incubated at 5% CO_2_, 37 °C for 1 h in the dark. Then, cells were treated with 5 μL of 12 × OMN and OMU EVOO extracts to reach the final concentrations of 1.0, 10.0, 50.0 and 100.0 µg/mL and incubated in the dark at 37 °C for 1 h. To induce ROS, cells were treated with H_2_O_2_ at a final concentration of 0.5 mM for 30 min a 37 °C in the dark and fluorescence signals (ex./ em. 490/525 nm) were recorded using Synergy H1 fluorescence plate reader (Biotek, Bad Friedrichshall, Germany).

### 2.12. Lipid Peroxidation (Malondialdehyde Equivalent, MDA Eq) Assay

HepG2 cells (2.5 × 10^5^ cells/well) were incubated with 10 and 25 μg/mL of OMN and OMU samples for 24 h at 37 °C under 5% CO_2_ atmosphere. The day after, cells were incubated with 1 mM H_2_O_2_ or vehicle (H_2_O) for 30 min, then collected and homogenized in 150 μL ice-cold MDA lysis buffer containing 1.5 μL of BHT (100×). Samples were centrifuged at 13,000× *g* for 10 min, then they were filtered through a 0.2 μm filter to remove insoluble material. To form the MDA- thiobarbituric acid (TBA) adduct, 300 μL of the TBA solution were added into each vial containing 100 µL of samples and incubated at 95 °C for 60 min, then cooled to RT for 10 min in an ice bath. For analysis, 100 μL of each reaction mixture were pipetted into a 96 well plate and the absorbance was measured at 532 nm using the Synergy H1 fluorescence plate reader (Biotek, Bad Friedrichshall, Germany).

### 2.13. 3-Hydroxy-3-Methylglutaryl Coenzyme a Reductase (HMGCoAR) Activity Assay

The experiments were carried out following the manufacturer’s instructions and conditions previously optimized [28] and more details are available on Supplementary Materials.

### 2.14. Western Blot Analysis

Western blot experiments have been performed using conditions previously optimized [29]. More details are available on Supplementary Materials.

### 2.15. In-Cell Western (ICW) Assay

Experiments have been performed using conditions previously optimized [30]. More details are available on Supplementary Materials.

### 2.16. Assay for the Evaluation of Fluorescent LDL Uptake by HepG2 Cells

Experiments have been performed using conditions previously optimized [31]. More details are available on Supplementary Materials.

### 2.17. Statistically Analysis

Statistical analyses were carried out by the t-student, One-way and Two-way ANOVA, and GraphPad Prism 8. Values were expressed as means ± standard deviation (s.d.); *p*-values < 0.05, were considered to be significant.

## 3. Results & Discussion

This work compares OMN and OMU samples obtained respectively with traditional methods in industrial mills equipped as described in materials and methods and with the use of ultrasound produced through the SHE prototype. The prototype was assembled and tested in the 2019 olive oil campaign and the design and operating characteristics are reported in literature [4,32].

### 3.1. Comparison of the VOCs Profile of the EVOO Samples

The analysis of the VOCs of EVOO samples provide useful information on the product quality, mainly from a sensory standpoint. The positive attributes are mainly associated to the VOCs originated from the lipoxygenase (LOX) pathway, whereas other classes of VOCs are associated to the main sensory defects (i.e., rancid, winey-vinegary, fusty/muddy sediment, musty/humidity/earthy, frostbitten olives/wet wood) [19,20,33,34].

Table 1 shows the amount of the main LOX-related VOCs in the two analyzed samples, but quite significant slight differences between the two samples were only observed for two VOCs (i.e., penten-3-one, E-2-hexenol and hexanal), confirming that the innovative extraction processes did not affect the profile of LOX-related VOCs. Accounting for almost 90% of the LOX-related VOCs content, E-2-hexenal was the most abundant component, according to available literature [20].

In order to evaluate the impact of the VOCs linked to the negative attributes, it was decided to calculate the marker recently proposed for the rancid defects, consisting of the sum of the content of pentanal, nonanal, and E-2-heptenal [20]. For both samples, the marker was equal to 0.025 mg/kg, much smaller than the limit value of 0.65 mg/kg, above which the sensation of rancid can be perceived [20].

Finally, the application of the chemometric approaches, recently proposed for supporting the Panel Test in Virgin Olive Oil classification [19], confirmed that both samples belong to the EVOO category.

### 3.2. Comparison of the Phenolic Profiles of the EVOO Samples

Similarly, the phenolic profiles of the two oils obtained before hydrolysis (IOC method) were very similar as well as the phenol contents determined after acidic hydrolysis (Table 2).

**Figure 1:**
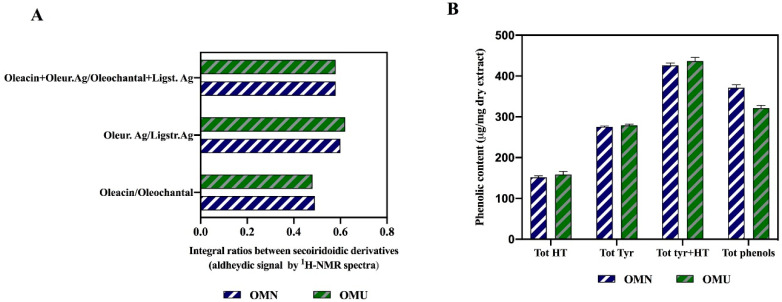
Analysis of secoiridoids. (**A**) Ratios of the integrals corresponding to the signals of secoiridoidic monoaldehydes in ^1^H-NMR spectra of the dry extracts from OMN and OMU samples. Oleur. Ag, aglycone of oleuropein; Ligstr. Ag. aglycone of ligstroside. (**B**) Total tyrosol + hydroxytyrosol (after hydrolysis) and phenolic content (according to the IOC method) in the dry extracts from OMN and OMU oil; each data represents the mean of the analysis of three extracts of the same samples.

The ^1^H^-^NMR spectra of the phenolic extracts allowed the evaluation of the ratios among the characteristic secoiridoids, measured as the aldehydes derived from the transformation during milling of the oleuropein and ligstroside precursors (Figure S1). This analysis showed that the ultrasound treatment did not change the profiles of the main secoiridoidic components of the phenolic fractions (Figure 1A). As for the dry extracts, the concentration of the total simple phenols (Tyr and OH-Tyr) and the total phenolic quantity evaluated before and after hydrolysis (Tyr + OH-Tyr) is shown in Figure 1B that hightlights the strong similarity of the two samples, with only a tiny difference between the total phenols before hydrolysis.

The use of ultrasounds has been already shown to increase oil yields while guaranteeing oil quality preservation. Several couples of EVOOs were analyzed and no significant differences were highlighted for their phenolic profile and volatiles produced by the lipoxygenase cascade [13].

### 3.3. Comparison of the In Vitro Antioxidant Activity of the Phenolic Extracts by DPPH and ABTS Assays

The correlation between the total phenol contents and the antioxidant activity has been widely studied in olive oil [35]. The antioxidant activity of an EVOO significantly increases in the presence of a high phenol concentrations. The genotype, the cultivation area and climate, and the extraction technique represent the major factors influencing the variability of the levels of phenolic compounds. This variability greatly affects not only the organoleptic, but also the nutraceutical features of each EVOO sample.

Different robust and reproducible methods for measuring the antioxidant capacity of olive oil are successfully and equally applied worldwide [36]. To evaluate the overall antioxidant activity of EVOO extracts, we decided to employ the DPPH and ABTS assays.

**Figure 2:**
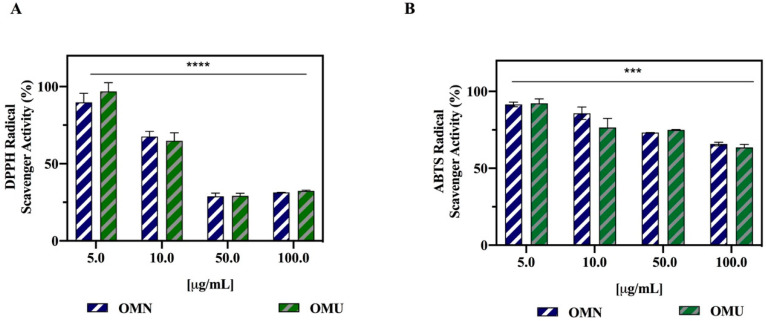
Antioxidant effects of OMN and OMU extracts. (**A**) In vitro radical scavenging activity of OMN and OMU phenol extracts by DPPH assay. (**B**) In vitro radical scavenging activity of OMN and OMU phenol extracts by ABTS assay. Data represent the mean ± s.d. of six determinations performed in triplicate. All the data sets have been analyzed by Two-way ANOVA. In particular, the reductions of DPPH (****) *p* < 0.0001 and ABTS (***) *p* < 0.001 radicals are significant as function of the concentrations, whereas no significant difference have been observed between OMN and OMU extracts.

The DPPH radical scavenging assay is one of the most commonly used single electron transfer (SET)-based antioxidant procedure due to its ease of performance, rapidness, automation potential, reproducibility, and usability at ambient temperatures [37]. Each extract was tested in the range of concentration from 5.0 to 100 µg/mL. The results clearly suggested that both OMU and OMN are able to scavenge the DPPH radical (Figure 2A). OMU diminished the DPPH radicals by 3.2 ± 5.6%, 35.2 ± 3.9%, 70.9 ± 1.4%, and 67.6 ± 0.4% at 5, 10, 50, and 100 µg/mL, respectively, whereas OMN reduced the DPPH radicals by 10.3 ± 5.4%, 32.4 ± 3.4%, 71.1 ± 1.2%, and 68.7 ± 0.1%, respectively, indicating that both extracts display similar radical scavenging activities. At the low dose of 5 µg/mL, the DPPH reduction was not significant. Starting from the same phenol-rich variety Coratina, the in vitro scavenging activities of the final products do not appear to be affected by the ultrasound process, in agreement with the chemical analyses (Table 1 and Table 2).

Hydroxytyrosol (HT) and oleuropein (Ole) are known as peroxy radical scavengers and are usually associated with the antioxidant activities of olive products [38]. In light with this information, additional experiments were performed in order to evaluate the in vitro radical scavenging activities of these compounds by performing the same DPPH assay (Figure S2). HT and Ole appear to be better antioxidants than Tyr (Figure S2), suggesting their active contribution to the scavenging activities of both OMN and OMU phytocomplexes. Similar results have been observed some years ago by Carrasco-Pancorbo and co-workers [39], who hypothesized that the lower antioxidant activity of Tyr compared to HT can be explained by the absence of the ortho-diphenolic group in its structure [40].

In parallel, each extract was assessed by using the ABTS. OMU diminished the ABTS radicals by 8.4 ± 1.4%, 14.2 ± 4%, 26.8 ± 0.1%, and 34.2 ± 1.1% at 5, 10, 50, and 100 µg/mL, respectively (Figure 2B), whereas OMU by 7.7 ± 2.9%, 23.5 ± 5.9%, 25 ± 0.3%, and 34.5 ± 2% at 5, 10, 50, and 100 µg/mL, respectively. Surprisingly, at the low concentration of 5 µg/mL, the ABTS reduction was significant for both extracts (* *p* < 0.5). Both phytocomplexes were therefore active, but with a smaller potency than in the DPPH assay. The differences between the DPPH and ABTS methods may be possibly ascribed to the different solvent used in these assays [41]. In fact, MeOH is used as solvent in the DPPH assay, whereas H_2_O is used in ABTS assay [42]. Considering that the phytocomplex had been extracted from the EVOO samples with a MeOH:H_2_O 80:20 v/v mixture, it seems possible to affirm that both OMN and OMU extracts are predominantly characterized by methanol soluble compounds. For instance, HT, which is more soluble in methanol, shows a better antioxidant activity in the DPPH than in the ABTS assay [43].

### 3.4. Comparison of the Antioxidant Effects on HepG2 Cells

Upon absorption from the gastrointestinal tract, the liver represents not only the main target for phenolic antioxidants, but also the major organ deputed to phenol metabolism [44,45]. Moreover, hepatocyte mitochondria and endoplasmic reticulum are the major sites for the generation of reactive oxygen species (ROS) in various forms of liver diseases [46]. The human hepatic HepG2 cell line was thus chosen here to compare the antioxidant activity of these samples. Indeed, HepG2, a well-differentiated transformed cell line, is a reliable model, easy to culture, well characterized, and widely used for biochemical and nutritional studies where many antioxidants and conditions can be assayed with minor inter-assay variations [47].

**Figure 3:**
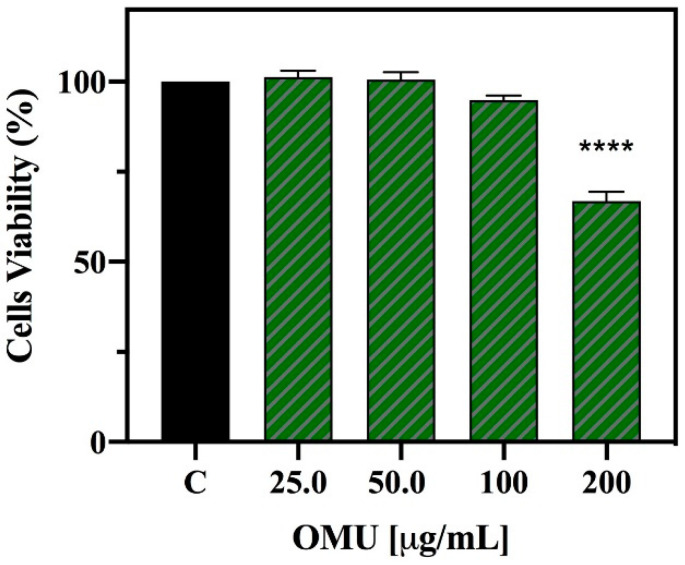
MTT assay. Effect of OMU extract on the HepG2 cell viability. Data represent the mean ± s.d. of three independent experiments performed in triplicate. The statistical significance of C vs OMU 200 µg/mL was analyzed by t-student test. (****) *p* < 0.0001, C: control cells.

Preliminary cell viability experiments were carried out using MTT assay in order to assess the concentrations of the OMU extract that may potentially determine cytotoxicity on HepG2 cells. After a 48 h treatment, no significant cell mortality had been detected up to 100 µg/mL versus untreated cells (C), whereas at 200 µg/mL a 33.1 ± 1.8% cell mortality was detected (Figure 3). In a similar experiment previously performed on OMN, no cytotoxic effect was detected up to 100 µg/mL versus untreated cells (C) [23].

**Figure 4:**
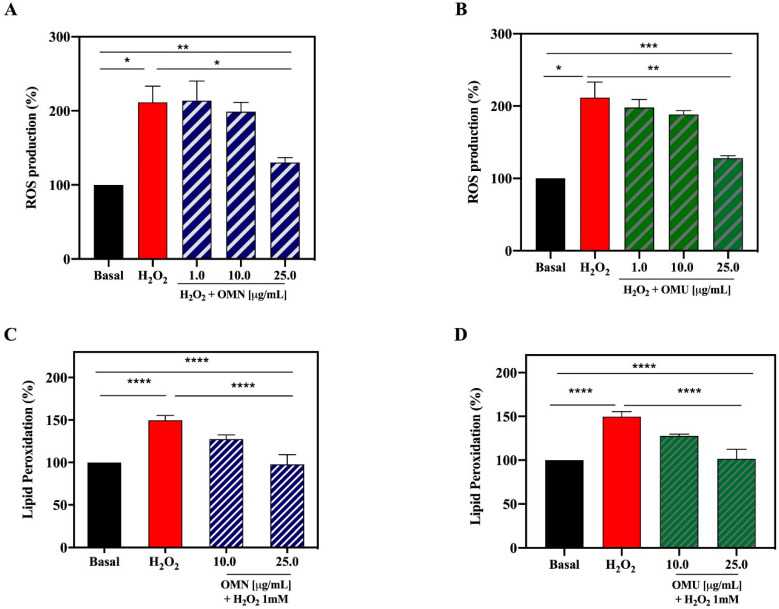
Antioxidant effects of OMN and OMU extracts on HepG2 cells. (**A**) OMN and (**B**) OMU reduce the H_2_O_2_ (1 mM)-induced ROS levels in HepG2 cells. (**C**) OMN and (**D**) OMU decrease the lipid peroxidation in the same cells after oxidative stress induction by H_2_O_2_. Data represent the mean ± s.d. of six independent experiments performed in triplicate. All the data sets were analyzed by One-way ANOVA; basal vs H_2_O_2_ samples were analyzed by t-student test, whereas H_2_O_2_ vs OMN/OMU + H_2_O_2_ samples by One-way ANOVA. (*) *p* < 0.5; (**) *p* < 0.01; (***) *p* < 0.001; (****) *p* < 0.0001.

In order to evaluate whether OMN and OMU extracts can modulate the H_2_O_2_-induced ROS production, HepG2 cells were pre-treated with both extracts (in the concentration range 1.0–25.0 µg/mL) at 37 °C overnight. The following day, the cells were treated with H_2_O_2_ (1.0 mM) at 37 °C for 30 min. Figure 4A,B clearly highlights that HepG2 cells, exposed to H_2_O_2_ alone, produce a dramatic increment of intracellular ROS levels by 211.4 ± 21.7% versus the control cells (basal value = 100%, *p* < 0.5), which was attenuated by the pre-treatment with OMN and OMU. OMN reduced the H_2_O_2_-induced intracellular ROS by 198.8 ± 12.5% and 130.3 ± 11.3% at 10.0 and 25.0 µg/mL, respectively (*p* < 0.01) (Figure 4A), whereas OMU by 197.9 ± 11.2, 188.5 ± 5.0, 128.0 ± 0.3 at 1.0, 10.0, and 25.0 µg/mL, respectively (*p* < 0.001) (Figure 4B).

These findings indicate that the pre-treatments with both extracts protect, in a similar way, the HepG2 cells against the increase of intracellular ROS induced by the H_2_O_2_ addition, thus restoring the ROS levels. These results are in agreement with a literate study on traditional EVOO samples performed using a similar protocol [48]. In addition, other studies have suggested that EVOO extracts impair the ROS generation induced by oxidative stress in other cellular systems, such as intestinal and muscle ones. The observed effects may depend mostly on HT that has been demonstrated to be able to reduce ROS generation induced by tert-butylhydroperoxide (t-BOOH) in HepG2 cells [47].

Lipids of cellular membranes are susceptible to oxidative attack, typically by ROS, resulting in a well-defined chain reaction with the production of end products, such as malondialdehyde (MDA) and related compounds, known as TBA reactive substances (TBARS). MDA is widely used as an index of lipoperoxidation in biological and medical sciences and elevated amounts of this metabolite have been found in various diseases linked to free radical damage [49]. Based on these considerations, the capacity of OMN and OMU to modulate the H_2_O_2_-induced lipid peroxidation in human hepatic HepG2 cells was assessed measuring the reaction of MDA precursor with the TBA reagent to form a fluorescent product (λex = 532/λem = 553 nm), proportional to the amount of TBARS (MDA equivalents) present. Figure 3 clearly suggests that, in agreement with the observed increase of ROS after the H_2_O_2_ treatment, a significant increase of the lipid peroxidation at cellular level up to 151.3 ± 6.6% was detected (*p* < 0.01). However, the pre-treatment of HepG2 cells with both EVOO extracts produce a significant reduction of lipid peroxidation even under basal conditions (*p* < 0.5) (Figure 4C,D). OMN decreases the lipid peroxidation up to 127.5 ± 5.3% and 97.8 ± 7.1% at 10 and 25 µg/mL, respectively, whereas OMU up to 127.7 ± 2.1% and 101.4 ± 10.3%, respectively, at the same concentrations. Again, any significant difference was observed as a function of the innovative EVOO extraction method.

Presumably, the reduction of lipid peroxidation induced by oxidative stress is mostly mediated by the contribution of both HT and Tyr in the EVOO extracts. In fact, clear evidence suggests that HT protects the integrity of the HepG2 cellular membrane leading to a reduction of MDA levels after t-BOOH induced oxidative stress [47]. Similarly, Tyr reduces lipid peroxidation in HepG2 cells exposed to acute ethanol treatment [50]. Other evidence confirms that Tyr has a protective effect on membrane integrity in other cellular systems. On the contrary, there are literature indications that the Ole-mediated protective effect against oxidative stress is not associated with a reduction of MDA generation [51].

### 3.5. Comparison of the Hypocholesterolemic Activity of the OMU and OMN Phenol Extracts

The beneficial effects of EVOO are linked to its ability to reduce the oxidative stress and to limit the lipoprotein oxidation, making the LDL less atherogenic. In fact, in patients with mild hypertension, the intake of EVOO improves the oxidation state of plasmatic LDL, decreases their peroxidation, increases the reduced glutathione, and decreases hypertension [52]. In a trial with 200 volunteers, the intake of EVOO led to an increase in high-density lipoproteins (HDL) and a simultaneous reduction of oxidation state of plasma [53]. Recently, we have disclosed a new molecular mechanism through which EVOO extracts may exhibit cholesterol-lowering activities by a directly modulation of the cholesterol biosynthetic pathway [23].

**Figure 5:**
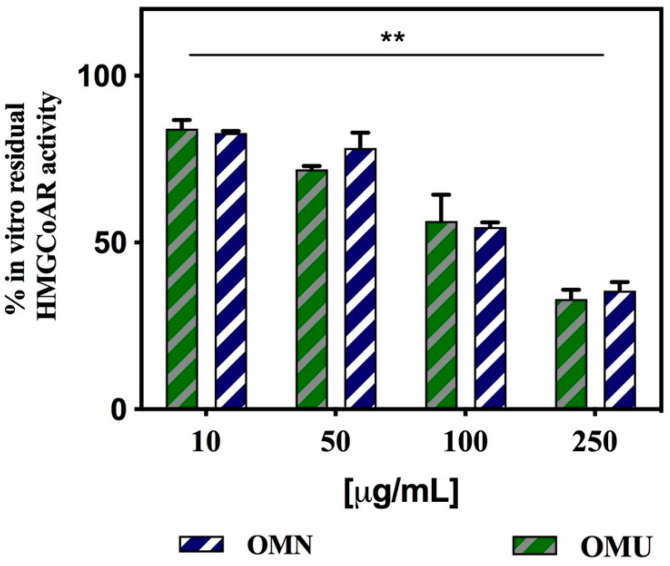
Effect of OMN and OMU extracts on the in vitro activity of HMGCoAR. Data represent the mean ± s.d. of three determinations performed in triplicate. All the data sets were analyzed by two-way ANOVA. In particular, the reduction of enzyme activity is significant as function of the all the tested concentrations (**) *p* < 0.01, whereas no significant difference was observed between OMN and OMU extracts.

These innovative and groundbreaking results prompt us to assess the capacity of OMU extract to modulate the HMGCoAR activity (Figure 5). To achieve this goal, in vitro experiments were carried out using the purified recombinant catalytic domain of the enzyme. Testing OMU extract, the residual enzyme activity was 84.2 ± 2.5%, 71.9 ± 1.1%, 56.4 ± 7.9%, and 33.0 ± 2.8% respectively at the concentrations of 10.0, 50.0, 100.0, and 250.0 µg/mL (Figure 5, green bars). Similarly, testing OMN, as the reference extract, the residual in vitro HMGCoAR activity was 82.5 ± 0.7%, 78.5 ± 4.6%, 54.8 ± 1.4%, and 35.6 ± 2.6%, respectively in the same range of concentrations (Figure 5, blue bars). A statistical analysis performed by two-way ANOVA indicated that a significant difference in the residual activity of the enzyme was observed as a function of the tested concentrations for both OMN and OMU (** *p* < 0.01), whereas any significant difference was observed between OMN and OMU extract at each tested dose. These results are also in line with those previously obtained for the reference OMN extract [23]. These results indicate that the new ultrasound process does not impair the molecular mechanism through which EVOO extracts promote hypocholesterolemic effect through the modulation of HMGCoAR activity, an enzyme that is crucial in cholesterol biosynthesis and is also the well-known target of statins [45,54].

In order to assess the role of the main components of the phytocomplex, we investigated in parallel Ole, Tyr and HT alone (Figure S3). Ole and Tyr were ineffective, whereas a slightly inhibition was observed with HT only at the concentration of 100 µM. These results underline the unique feature of the EVOO extracts, excluding the predominant role of any among Ole, Tyr, and HT when tested alone in the modulation of the enzyme activity. Thus, all these results clearly suggest that a synergistic contribution of the total components of phytocomplex is responsible for the inhibition of the HMGCoAR activity.

**Figure 6:**
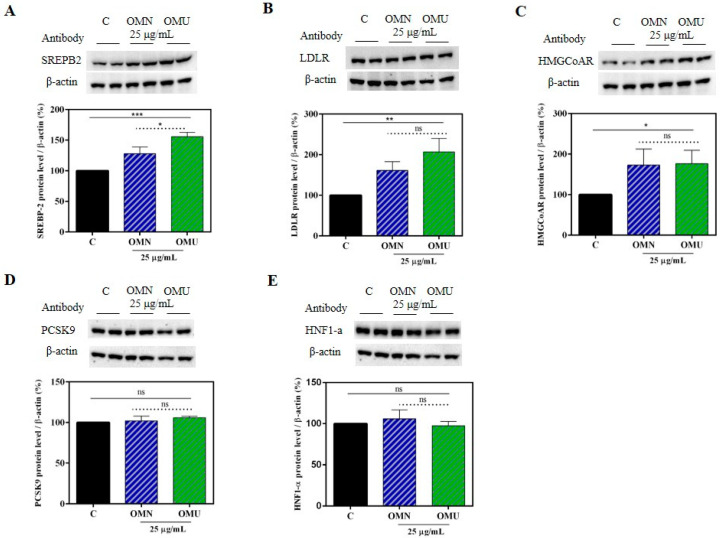
Modulation of cholesterol biosynthesis. (**A**) Western blot of SREBP-2 (precursor); (**B**) western blot of the LDLR; (**C**) western blot of HMGCoAR; (**D**) western blot of PCSK9; (**E**) western blot of HNF1- α. Data represent the mean ± s.d. of eight independent experiments performed in duplicate. All the data sets were analyzed by One-way ANOVA and OMN vs OMU by t-student test. (*) *p* < 0.5; (**) *p* < 0.01; (***) *p* < 0.001; ns: not significant. C: control sample.

On this basis, we decided to compare the ability of these extracts to modulate the LDLR pathway. Thus, HepG2 cells were treated with 25.0 μg/mL of each extract (a doses that is about 10 folds lower than the smallest cytotoxic dose and a compromise between it and the effect on the in vitro HMGCoAR activity). The western blot experiments, assessed on cell lysates, showed that LDLR pathway was activated after OMN and OMU treatments (Figure 6A–C). The OMU and OMN extracts increased the protein level of the sterol regulatory element-binding transcription factor (SREBP)-2 (precursor) by 156 ± 6.8% and 134% ± 10.9% (*p* < 0.001), respectively (Figure 6A), which in turn led to an augmentation of LDLR protein levels up to 207 ± 33% and 161 ± 21.7% (*p* < 0.01), respectively (Figure 6B). Upon the activation of the SREBP-2 transcription factor, improvements of HMGCoAR protein levels were also observed, by 158 ± 33% with OMU and by 153 ± 39.8% (*p* < 0.05) with OMN (Figure 6C).

The LDLR is a key player in the cholesterol metabolism pathway. In fact, it is regulated at transcriptional, translational, and post-translational levels. In particular, the dynamic trafficking of functional LDLR is finely regulated by a protein named proprotein convertase subtilisin/kexin type 9 (PCSK9). Both SREBP-2 and hepatocyte nuclear factor 1-alpha (HNF1-α) cooperatively transcriptional activate the PCSK9 gene expression, but only SREBP-2 controls the LDLR expression. In this context, statins enhance the level of PCSK9 leading to an improvement of LDLR degradation.

Herein, the contextual modulation of PCSK9 intracellular processing was also investigated in HepG2 cells. OMU and OMN did not modulate the mature PCSK9 protein levels (Figure 6D) and to activate HNF1-α, the PCSK9 transcription factor (Figure 6E). This result reinforces the main outcome of a previous work [23], which has suggested that EVOO phenols are able to improve the cholesterol metabolism pathway without increasing the PCSK9 protein levels, a relevant drawback of statins [55].

**Figure 7:**
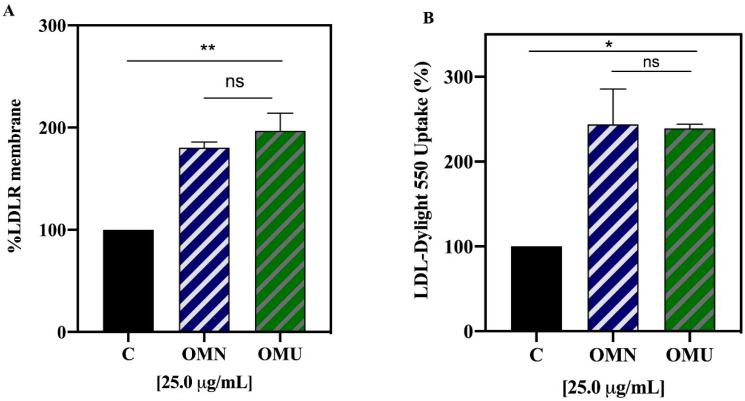
Modulation of the LDLR on HepG2 cell surface and uptake of environmental LDL. (**A**) LDLR protein levels on HepG2 cell surface evaluated by in cell western. (**B**) Uptake of fluorescent LDL from the environment by HepG2 cells. Data represent the mean ± s.d. of five independent experiments performed in triplicate. All the data sets were analyzed by One-way ANOVA and OMN vs OMU by t-student test. (*) *p* < 0.5; (**) *p* < 0.001. ns: not significant, C: control sample.

In order to compare the ability of the extracts to modulate the levels of the LDLR population localized on the hepatocyte surface, a specific ICW assay was performed in parallel on both extracts tested at 25 µg/mL. This cell-based assay permits the target protein detections in fixed cultured cells. Improvements of the membrane LDLR levels by 197 ± 5.3 % for OMU and by 180 ± 17.2% for OMN extracts were observed (Figure 7A). Also in this case, no significant differences were detected between the two samples.

Finally, functional experiments were carried out to compare the effects of the two extracts on the capacity of HepG2 cells to uptake the LDL from the extracellular environment. The experiment (conducted at 25.0 µg/mL) showed that the ability of HepG2 cells to absorb fluorescent LDL from the extracellular space was increased by 239.3 ± 34.2% by OMU and by 243.9 ± 6.5 % by OMN (Figure 7B). Therefore, even here there are only negligible differences in the capacity of the two extracts to stimulate the uptake of the LDL by HepG2 cells.

Indeed, these results show that through the inhibition of the HMGCoAR activity, both OMN and OMU extracts modulate the cellular cholesterol metabolism, leading to the LDLR and HMGCoAR protein level augmentations by increasing the SREBP-2 (precursor) protein levels (Figure 6). In addition, with a similar behavior, both extracts increase the population of LDLR which are localized on the membrane of HepG2 cells, which leads from a functional point of view to an increased ability of HepG2 cells to absorb extracellular LDL, with an in vitro cholesterol-lowering effect (Figure 7). The membrane LDLR activity is also positively regulated by the fact that both OMN and OMU extracts did not produce any variation of PCSK9 protein levels, clearly confirming that similarly to OMN, also OMU shows a distinct and unique behavior in respect to statins.

## 4. Conclusions

Performing a series of chemical, biochemical and cellular investigations, in this study we have demonstrated the substantial equivalence of the EVOO sample OMU, produced with an innovative process based on the simultaneous treatment of olive paste both with ultrasound and heat-exchange, and the sample OMN produced with the traditional methodology. This means that the increase of the yield has not impaired any relevant feature of the EVOO, neither on the standpoint of the chemical composition and sensory characteristics nor the nutraceutical properties, as far as the antioxidant and hypocholesterolemic activities are involved.

Properly communicated, these results may represent a tool for reducing the neophobia linked to the introduction of a relevant innovation in a traditional food sector such as the EVOO production. The possibility of guaranteeing a price premium to this kind of product may assure a greater environmental and economic sustainability for stakeholders in the supply chain, achieving the European goal of improving simultaneously industry competitiveness, natural resources protection, and citizen health.

## Figures and Tables

**Table 1:** Profile of the lipoxygenase LOX-related volatile organic compounds VOCs in the two analyzed samples. Results are expressed as mean of triplicate measurements. For each VOC, different letters indicate significant differences between the two samples. EVOO from cultivar Coratina with (**OMU**) or without (**OMN**) using the ultrasound technology

VOC (mg/kg)	OMU	OMN
penten-3-one	0.504 a	0.634 b
*E*-2-pentenal	0.043 a	0.048 a
penten-3-ol	0.854 a	0.876 a
*Z*-3-hexenal	<LOD	<LOD
*E*-2-hexenal	33.889 a	33.160 a
*E*-2-pentenol	0.058 a	0.054 a
*Z*-2-pentenol	0.023 a	0.024 a
*Z*-3-Hexenyl acetate	0.053 a	0.054 a
*Z*-3-hexenol	0.467 a	0.433 a
*E*-2-hexenol	0.731 a	0.678 a
Hexanal	0.886 a	1.081 b
Hexyl acetate	0.010 a	0.009 a
Hexanol	0.570 a	0.516 a
ƩLOX	38.090 a	37.569 a

**Table 2:** Phenol contents determined before and after acidic hydrolysis. Results are expressed as mean ± s.d. of analysis of three extracts of the same samples.

Phenol Contents in the Oils (mg/kg)				
	(Before Hydrolysis)		(After Hydrolysis)	
	OMN	OMU	OMN	OMU
**Free hydroxytyrosol**	17.4 ± 5.1	18.1 ±2.6	169.6 ± 2.6	185.1 ± 13.4
**Free tyrosol**	15.6 ± 5.4	14.3 ± 3.0	308.6 ± 2.7	325.7 ±23.4
**Total Phenols**	415.7 ± 7.3	375.1 ± 8.5	478.1 ± 4.7	510.7 ± 36.7

## Supplementary Materials

The following are available online at https://www.mdpi.com/2076-3921/9/9/798/s1, Description of Material and Methods, Figure S1. ^1^H-NMR spectra of the phenolic extracts from OMN and OMU. Profiles of the main secoiridoidic components of the phenolic fraction, Figure S2. Antioxidant activity evaluation of (**A**) hydroxytyrosol (HT), (**B**) Oleuropein (Ole), and (**C**) Tyrosol (Tyr) by DPPH assay in the range of concentration 10–250 µM, Figure S3. Effect of HT, Ole, and Tyr on the in vitro HMGCoaR activity.
