# Supplementary material for: Virgin Olive Oil Extracts Reduce Oxidative Stress and Modulate Cholesterol Metabolism: Comparison between Oils Obtained with Traditional and Innovative Processes
Source: Antioxidants (Basel). 2020 Aug 27;9(9):798. doi: 10.3390/antiox9090798 (PMC7555338; doi:10.3390/antiox9090798)
Supplement: Supplementary file 1 [file antioxidants-09-00798-s001.pdf]

Supplementary Materials

Virgin Olive Oil Extracts reduce Oxidative Stress and modulate Cholesterol Metabolism: Comparison between Oils obtained with Traditional and Innovative Processes

Carmen Lammi[1*], Nadia Mulinacci[2], Lorenzo Cecchi[2], Maria Bellumori[2], Carlotta Bollati[1], Martina Bartolomei[1], Carlo Franchini[4], Maria Lisa Clodoveo[3], Filomena Corbo[4], Anna Arnoldi[1].

1. Department of Pharmaceutical Sciences, University of Milan, 20133 Milan, Italy.
2. Department of Neuroscience, Psychology, Drug and Child Health, Pharmaceutical and Nutraceutical Section, University of Florence, 50019 Florence, Italy.
3. Interdisciplinar Department of Medicine University Aldo Moro Bari, 70125 Bari, Italy
4. Department of Pharmacy-Pharmaceutical Sciences, University Aldo Moro Bari, 70125 Bari, Italy

* Correspondence: carmen.lammi@unimi.it; Tel +39-025-031-9372

Materials and Methods

2.1 Chemicals

Dulbecco's modified Eagle's medium (DMEM), L-glutamine, fetal bovine serum (FBS), phosphate buffered saline (PBS), penicillin/streptomycin, chemiluminescent reagent, and 24 or 96-well plates were purchased from Euroclone (Milan, Italy). MTT [3-(4,5-dimethylthiazol-2-yl)-2,5-diphenyltetrazolium bromide], DPPH (1,1-diphenyl-2-picrylhydrazyl), Janus Green, formaldehyde, HCl, H_2SO_4, bovine serum albumin (BSA), RIPA buffer, the antibody against β-actin, fluorometric intracellular ROS kit and HMGCoAR assay kit were bought from Sigma-Aldrich (St. Louis, MO, USA). The antibody against HMGCoAR was bought from Abcam (Cambridge, UK). Phenylmethanesulfonyl fluoride (PMSF), Na-orthovanadate inhibitors, and the antibodies against rabbit Ig-horseradish peroxidase (HRP), mouse Ig-HRP, and SREBP-2 [which recognizes epitope located in a region between 833-1141 and bands at about 132 kDa] were purchased from Santa Cruz Biotechnology Inc. (Santa Cruz, CA, USA). The antibody against the low-density lipoprotein receptor (LDLR) was bought from Pierce (Rockford, IL, USA). The antibodies against hepatocyte nuclear factor 1-alpha (HNF1-alpha) and proprotein convertase subtilisin/kexin type 9 (PCSK9) were bought from GeneTex (Irvine, CA, USA); the inhibitor cocktail Complete Midi from Roche (Basel, Swiss); Mini protean TGX pre-cast gel 7.5% and Mini nitrocellulose Transfer Packs from BioRad (Hercules, CA, USA); the LDL-DyLight™ 550 from Cayman Chemical (Michigan, USA).

2.9 Cell culture conditions and treatments

HepG2 cells, purchased from ATCC (HB-8065, ATCC from LGC Standards, Milan, Italy), were cultured in DMEM high glucose with stable L-glutamine, supplemented with 10% FBS, 100 U/mL penicillin, 100 μg/mL streptomycin (complete growth medium) with incubation at 37 °C under 5% CO_2 atmosphere. HepG2 cells were used for no more than 20 passages after thawing, because the increase in number of passages may change the cell characteristics and impair assay results.

OMN and OMU extracts were tested separately. Briefly, each EVOO extract was dissolved in DMSO in order to prepare stock solutions (50.0 mg/mL), which were diluted in order to reach the final concentration of 25.0 μg/mL in complete growth DMEM. The final 0.05% concentration of DMSO was kept constant either in treated or control cells. The growth medium of adherent HepG2 cells was discarded and each diluted EVOO extract in complete DMEM was replaced and incubated for the desirable incubation time based on the experiments.

2.10 3-(4,5-dimethylthiazol-2-yl)-2,5-diphenyltetrazolium bromide (MTT) assay

A total of 3 × 10^4 HepG2 cells/well were seeded in 96-well plates and treated with 25.0, 50.0, 100.0 and 200.0 μg/mL of OMN and OMU EVOO extracts, or vehicle (H_2O) in complete growth media for 48 h at 37 °C under 5% CO_2 atmosphere. Subsequently, the treatment solvent was aspirated and 100 μL/well of 3-(4,5-dimethylthiazol-2-yl)-2,5-diphenyltetrazolium bromide (MTT) filtered solution added. After 2 h of incubation at 37 °C under 5% CO_2 atmosphere, 0.5 mg/mL solution was aspirated and 100 μL/well of the lysis buffer (8 mM HCl + 0.5% NP-40 in DMSO) added. After 5 min of slow shaking, the absorbance at 575 nm was read on the Synergy H1 fluorescence plate reader (Biotek, Bad Friedrichshall, Germany).

2.13 HMGCoAR activity assay

The assay buffer, NADPH, substrate solution, and HMGCoAR were provided in the HMGCoAR Assay Kit (Sigma). The experiments were carried out following the manufacturer's instructions and conditions previously optimized [28] at 37 °C. Each reaction (200 μL) was prepared adding the reagents in the following order: 1 X assay buffer, pravastatin (1.0 μM) or OMN and OMU EVOO extracts (10- 250 μg/mL) or vehicle (C), the NADPH (4 μL), the substrate solution (12 μL), and finally the HMGCoAR (catalytic domain) (2 μL). Subsequently, the samples were mixed and the absorbance at 340 nm read by a microplate reader Synergy H1 from Biotek at time 0 and 10 min. The HMGCoAR-dependent oxidation of NADPH and the inhibition properties of EVOO extracts were measured by absorbance reduction, which is directly proportional to enzyme activity

2.14 Western blot analysis

Briefly, HepG2 cells/well (1.5 × 10^5 cells/wel 24-well plate) were treated with OMN and OMU samples (25.0 μg/mL) for 24 h. After each treatment, cells were scraped in 30 μL ice-cold lysis buffer [RIPA buffer + inhibitor cocktail + 1:100 PMSF + 1:100 Na-orthovanadate] and transferred in an ice-cold microcentrifuge tube. After centrifugation at 13,300g for 15 min at 4 °C, the supernatant was recovered and transferred into a new ice-cold tube. Total proteins were quantified by Bradford method and 50 μg of total proteins loaded on a pre-cast 7.5% Sodium Dodecyl Sulfate - Polyacrylamide (SDS-PAGE) gel at 130 V for 45 min. Subsequently, the gel was pre-equilibrated with 0.04% SDS in H_2O for 15 min at RT and transferred to a nitrocellulose membrane (Mini nitrocellulose Transfer Packs,) using a trans-Blot Turbo at 1.3 A, 25 V for 7 min. Target proteins, on milk or BSA blocked membrane, were detected by primary antibodies as follows: anti-SREBP2, anti-LDLR, anti-HMGCoAR, anti-PCSK9, anti-HNF1-α, and anti-β-actin. Secondary antibodies conjugated with HRP and a chemiluminescent reagent were used to visualize target proteins and their signal was quantified using the Image Lab Software (Biorad). The internal control β-actin was used to normalize loading variations.

2.15 In-Cell Western (ICW) assay

A total of 3 × 10^4 HepG2 cells/well were seeded in 96-well plate and, the following day, they were treated with 25 μg/mL of OMN and OMU EVOO extracts in complete growth medium for 24 h. Subsequently, they were fixed in 4% paraformaldehyde for 20 min at RT. Cells were washed 5 times with 100 μL of PBS/well (each wash was for 5 min at RT) and the endogenous peroxides activity quenched adding 3% H_2O_2 for 20 min at RT. Non-specific sites were blocked with 100 μL/well of 5% bovine serum albumin (BSA, Sigma) in PBS for 1.5 h at RT. LDLR primary antibody solution (Abcam) (1:3000 in 5% BSA in PBS, 25 μL/well) was incubated O/N at +4 °C. Subsequently, the primary antibody solution was discarded, and each sample was washed 5 times with 100 μL/well of PBS (each wash was for 5 min at RT). Goat anti-rabbit Ig-HRP secondary antibody (Santa Cruz) solution (1:6000 in 5% BSA in PBS, 50μL/well) was added and incubated 1 h at RT. The secondary antibody solution was washed 5 times with 100 μL/well of PBS (each wash for 5 min at RT). Fresh prepared TMB Substrate (Pierce, 100 μL/well) was added and the plate was incubated at RT until the desired color was developed. The reaction was stopped with 2 M H_2SO_4 and then the absorbance at 450 nm was measured using a microplate reader Synergy H1 from Biotek. Cells were stained by adding 1 × Janus green stain, incubating for 5 min at RT. The dye was removed, and the sample washed 5 times with water. Afterward, 0.1 mL 0.5 M HCl per well were added and incubated for 10 min. After 10 s shaking, the OD at 595 nm was measured using the Synergy H1 fluorescent plate reader from Biotek.

2.16 Assay for the evaluation of fluorescent LDL uptake by HepG2 cells

Experiments have been performed using conditions previously optimized.[31] Briefly, a total of 3 × 10^4 HepG2 cells/well were seeded in 96-well plates and kept in complete growth medium for 2 days before treatment. On the third day, cells were treated with 25 μg/mL of OMN and OMU extracts or vehicle (H_2O) for 24 h. At the end of the treatment period, the culture medium was replaced with 50 μL/well LDL-DyLight™ 550 working solution. The cells were additionally incubated for 2 h at 37 °C and then the culture medium was aspirated and replaced with PBS (100 μL/well). The degree of LDL uptake was measured using the Synergy H1 fluorescent plate reader from Biotek (excitation and emission wavelengths 540 and 570 nm, respectively).

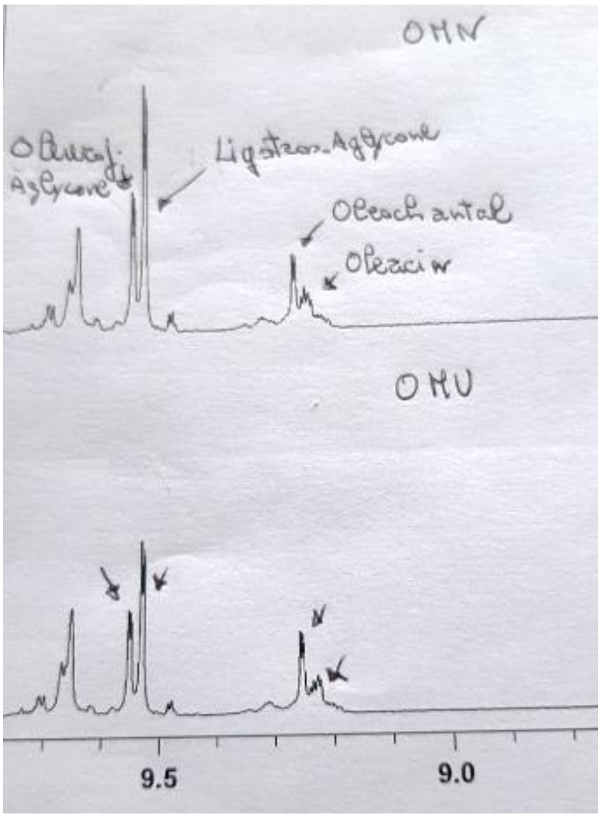

Figure S1. ^{1}H-NMR spectra of the phenolic extracts from OMN and OMU. Profiles of the main secoiridoidic components of the phenolic fraction

In vitro radical scavenging activity of of hydroxytyrosol (HT), Oleuropein (Ole), and Tyrosol (Tyr) by DPPH assay.

Each compound was separately tested in the range of concentration 10-250 µM. The results clearly suggested that only HT and Ole but not Tyr are able to scavenge the DPPH radical (Figure 1S). HT diminished the DPPH radicals by 8.7±2,4%, 44.8±1.7%, 82.0±0.5%, and 86.7±0.3% at 10, 50, 1100, and 250 µM, respectively, whereas Ole reduced the DPPH radicals by 7.1±4.4%, 29.7±6.0%, 54.9±0.1%, and 86.8±0.3%, respectively.

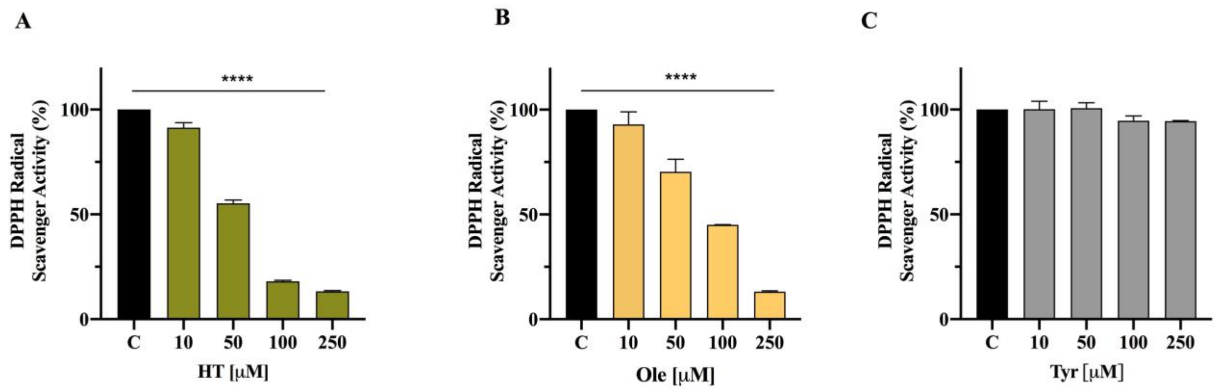

Figure S2. Atioxidant activity evaluation of (**A**) hydroxytyrosol (HT), (**B**) Oleuropein (Ole), and (**C**) Tyrosol (Tyr) by DPPH assay in the range of concentration 10-250 µM.

In vitro Effect of HT, Ole and Tyr on the HMGCoAR activity.

Each compound was assessed in order to evaluate their effect on the HMGCoAR activity. Results suggest that only HT and Ole are slightly active at 15.4 and 54 µg/mL, which correspond to 100 µM, respectively, whereas Tyr is totally ineffective at all the tested concentrations. In particular, HT reduces the enzyme activity by 25±4.5% and Ole 14.3±7.1% at 100 µM, respectively.

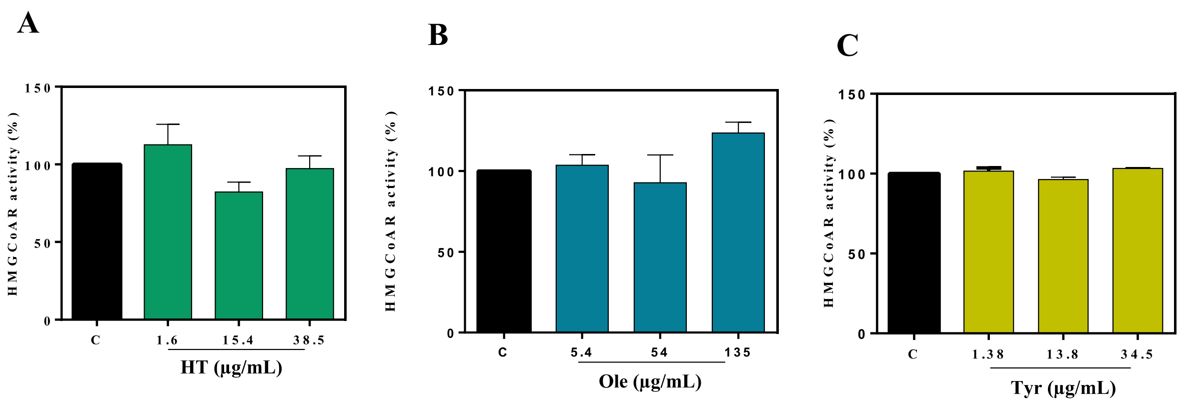

Figure S3. Effect of HT, Ole, and Tyr on the in vitro HMGCoaR activity.
